# ECM Mechanoregulation in Malignant Pleural Mesothelioma

**DOI:** 10.3389/fbioe.2022.797900

**Published:** 2022-02-14

**Authors:** Valeria Panzetta, Ida Musella, Sabato Fusco, Paolo A. Netti

**Affiliations:** ^1^ Department of Chemical, Materials and Production Engineering, University of Naples Federico II, Naples, Italy; ^2^ Centro di Ricerca Interdipartimentale sui Biomateriali CRIB, University of Naples Federico II, Naples, Italy; ^3^ Istituto Italiano di Tecnologia, IIT@CRIB, Naples, Italy; ^4^ Department of Medicine and Health Sciences “V. Tiberio”, University of Molise, Campobasso, Italy

**Keywords:** mechanobiology, mechanical properties of tumor tissues, cell biophysical properties, malignant pleural mesothelioma, atomic force microscope

## Abstract

Malignant pleural mesothelioma is a relatively rare, but devastating tumor, because of the difficulties in providing early diagnosis and effective treatments with conventional chemo- and radiotherapies. Patients usually present pleural effusions that can be used for diagnostic purposes by cytological analysis. This effusion cytology may take weeks or months to establish and has a limited sensitivity (30%–60%). Then, it is becoming increasingly urgent to develop alternative investigative methods to support the diagnosis of mesothelioma at an early stage when this cancer can be treated successfully. To this purpose, mechanobiology provides novel perspectives into the study of tumor onset and progression and new diagnostic tools for the mechanical characterization of tumor tissues. Here, we report a mechanical and biophysical characterization of malignant pleural mesothelioma cells as additional support to the diagnosis of pleural effusions. In particular, we examined a normal mesothelial cell line (Met5A) and two epithelioid mesothelioma cell lines (REN and MPP89), investigating how malignant transformation can influence cellular function like proliferation, cell migration, and cell spreading area with respect to the normal ones. These alterations also correlated with variations in cytoskeletal mechanical properties that, in turn, were measured on substrates mimicking the stiffness of patho-physiological ECM.

## Introduction

An increasing number of studies report on how the recognition of interplays between microenvironment and cell mechanics could reveal pathophysiological disfunctions at different hierarchical tissue levels. As a consequence, mechanical phenotyping of cell and extracellular matrix (ECM) is increasingly becoming a feasible and promising opportunity to strengthen the diagnosis process by supporting the clinical decision-making. In particular, it has been observed that the maintenance of the cell mechanical homeostasis, mainly related to the cytoskeleton, is fundamental to guarantee the physiological cellular functions (Paszek et al., 2005; Humphrey et al., 2014; Eichinger et al., 2021). The cytoskeleton of living cells, constantly remodeled by the complex interactions between cell and surrounding microenvironment, is a highly dynamic structure that regulates many cellular functions, such as adhesion, proliferation, migration, and differentiation (Alberts et al., 2002; Gardel et al., 2010; Parsons et al., 2010; Provenzano and Keely, 2011; Ambriz et al., 2018). Abnormalities in cytoskeleton dynamics contribute to the emergence and the progression of a large variety of pathological processes, such as cardiovascular diseases (Hein, 2000), neurodegenerative diseases (Podoga and Janmey, 2018; Viji Babu and Radmacher, 2019), and cancer (Hall, 2009; Fife et al., 2014). In particular, in the case of cancer, alterations in cell cytoskeleton are connected to changes in cell stiffness that entailed a softening of tumor cells in comparison with benign ones (Guck et al., 2005; Lekka et al., 2012; Lin et al., 2015; Panzetta et al., 2017a). Changes in the cytoskeletal structure also affect cell proliferation, adhesion, ability to remodel the surrounding matrix, and migration (Ingber et al., 1994; Alberts et al., 2002). The strong correlation existing between cell stiffness and cell malignancy allowed us to use cell mechanical properties as a new biomarker, not only to distinguish malignant from benign cells, but also to discriminate between cancer cells with different aggressive potential (Cross et al., 2007; Cross et al., 2008; Xu et al., 2012; Panzetta et al., 2017a). Recently, to deeply investigate the malignant transformation process from a mechanical point of view, it is also necessary to consider the biophysical crosstalk between cells and their surrounding environment. Through the cytoskeleton, the cells can sense the mechanical state of the surrounding ECM (Panzetta et al., 2019). In fact, through the formation of the adhesion structures at the cell–ECM interface, cells pulling and pushing on their surroundings remodel their microenvironment thanks to actomyosin- and cytoskeletal-generated forces that, in turn, adapt themselves to biophysical cues and the mechanical state of ECM (Panzetta et al., 2017b). Such mechanism is deeply regulatory for cell behavior and, consequently, loss of the correct mechanical crosstalk between cells and extracellular environment could promote pathological imbalances. Thus, it is essential to consider in which way changes in biophysical signals and mechanical properties of matrix could influence the cancer journey from genesis to invasion.

Malignant pleural mesothelioma (MPM) is a rare and highly aggressive disease that develops in the thin layer of the tissue surrounding the lungs, known as pleura. Asbestos exposure is considered the major cause of this disease and the long interval between exposure and the development of mesothelioma has been the reason for the relatively late discovery of the cause (Baas et al., 1998). Malignant mesothelioma results from the neoplastic transformation of mesothelial cells and is associated with phenotypic modifications and genetic changes that alter cell–cell and cell–matrix interactions and regulation of cell proliferation and cell death (Jean et al., 2012; Mossman et al., 2013; Sekido 2013). Patients often present with a pleural effusion that can be used for diagnostic purposes. Currently, the diagnosis of malignant pleural effusion relies on cytological analysis of pleural fluid. However, effusion cytology may take weeks or months to establish and has limited sensitivity (30%–60%) for diagnosing MPM (Han et al., 2013). For patients with inconclusive results following cytological analysis of the pleural fluid, the next step is a thoracoscopic pleural biopsy, which is an invasive procedure that requires a skilled operator. The interest in this relatively rare but devastating tumor arises from the fact that its incidence is increasing worldwide. MPM remains a challenge for pathologists and clinicians to treat because of difficulties in early diagnosis and resistance to conventional therapies. For this reason, alternative investigative methods are necessary to support the diagnosis of pleural effusion.

In this frame, many techniques of noticeable capability are being developed to probe cellular properties at the single-cell level directly on living cells (Fodil et al., 2003; Li et al., 2009; Kirmizis and Logothetidis, 2010; Zhou et al., 2010). Among these, atomic force microscopy (AFM) enables one to quantify the elastic modulus (Young’s modulus) of single cells in conditions close to the natural environment. AFM has rapidly become a valuable tool also to discriminate cancer cells with a different metastatic potential (Faria et al., 2008; Park and Lee, 2014) and to identify nanomechanical fingerprints of tumor tissue at both cell and ECM scale level for tumor prognosis and classification (Plodinec et al., 2012; Stylianou et al., 2018).

Here, we report a mechanical and biophysical characterization of MPM cells. We examined a normal mesothelial cell line (MET5a) and two epithelioid MPM cell lines (REN and MPP89), focusing our attention on which way the malignant transformation influences cell proliferation, cell migration, and cell spreading area with respect to the normal ones. These alterations were associated to variation in cytoskeletal mechanical properties, by AFM cell lines’ mechanical phenotyping. Finally, the influence of substrate stiffness on cell mechanics was investigated, considering that, as for the most of solid tumors, MPM is also accompanied by a stiffening and thickening process of the pleura because of the progressive scarring of the lung tissues caused by asbestos exposure (Wellman et al., 2018). At this aim, the different cell lines were cultured on polyacrylamide (PAAm) gels of different stiffness. Synthetic polymers, like PAAm, are selected because they do not interfere with microscopy thanks to their optical transparency and provide key features of the cell environment (Drury and Mooney, 2003; Ventre et al., 2012). Moreover, the elastic modulus of PAAm gels can be easily tuned by varying the cross-linker concentration.

## Materials and Methods

### Polyacrylamide Substrates Preparation and Mechanical Characterization

PAAm substrates were prepared by mixing acrylamide, methylene-bis-acrylamide, 1/100 total volume of 10% ammonium persulfate, and 1/1,000 total volume of N,N,N′,N′-tetramethylethylenediamide (TEMED). Different combinations of acrylamide and bis-acrylamide were used to obtain 0.3 kPa (3 wt/vol% acrylamide and 0.04 wt/vol% bis-acrylamide), 4 kPa (6 wt/vol% acrylamide and 0.06 wt/vol% bis-acrylamide), 13 kPa (10 wt/vol% acrylamide and 0.06 wt/vol% bis-acrylamide), and 30 kPa (10 wt/vol% acrylamide and 0.3 wt/vol% bis-acrylamide) hydrogels. To allow for cell adhesion, substrates were functionalized with collagen, by using a bifunctional photo-linker, N-sulfosuccinimidyl-6-(4′-azido-2′-nitrophenylamino) hexanoate (sulfo-SANPAH), as a cross-linking agent to immobilize collagen. The freshly prepared sulfo-SANPAH solution at a concentration of 0.2 mg/ml was placed onto PAAm substrates and exposed to UV light for 10 min. After washing with phosphate buffer saline (PBS, Microtech), the hydrogels were coated with 50 μg/ml of bovine type I collagen overnight at room temperature (RT). The mechanical properties of PAAm substrates were evaluated using a stress-controlled rheometer and a commercial AFM.

### Cell Culture

Experiments were performed on benign human mesothelial cells (Met5A) and two malignant human mesothelioma cells (REN and MPP89), with different metastatic potentials. Cell lines were cultured in RPMI 1640 (Microtech) supplemented with 10% fetal bovine serum (FBS, BioWhatter, MD), 2 mM L-glutamine (Sigma, St. Louis, MO), 1,000 U/L penicillin (Sigma, St. Louis, MO), and 100 mg/L streptomycin (Sigma, St. Louis, MO).

### Cell Proliferation and Migration

In proliferation experiments, cells (5 × 10^4^/well) were seeded in 6-well plates. Cell aliquots were collected at 24 and 48 h and were counted after 24 and 48 h from seeding. Cell proliferation was measured by counting cells in a Neubauer hemocytometer.

Single-cell and collective migration were investigated to study cell migratory behavior on both glass dishes and PAAm substrates. To examine single-cell migration, cells (2,000/cm^2^) were seeded and incubated at 37°C and 5% CO_2_ for 24 h to allow cell adhesion. After incubation, cell migration videos were recorded using Olympus IX81 inverted microscope at 4 × magnification, equipped with a digital camera (Hamamatsu, ORCA-Flash2.8). For each sample, ten regions were captured every 5 min for 12 h to allow the tracking of an average number between 100 and 200 cells. To study collective cell migration on glass dishes, the cell wound closure assay was used. Cells were seeded in 35-mm Petri dishes and incubated until confluence. Then, a scratch was made across the monolayer and the wound closure was recorded for 12 h, using the equipment previously described. The migration efficiency, expressed in terms of percentage of wound closure, was calculated by measuring three randomly chosen distances across the wound at four different time intervals (0, 4, 8, and 12 h). Furthermore, migration videos of single Met5A, REN and Mpp89 cells at the far ends of the wound were recorded using an Olympus IX81 inverted microscope at 10× magnification. Cell migration rate and directionality, defined as the ratio of accumulated distance (the sum of the distances of all trajectory vectors) to the Euclidean distance (distance between start and end points), were quantified. To evaluate if cell trajectories at the wound front exhibited similarities in their shape, the Pearson correlation coefficient (Pearson’s *r*) between the x(t) (Corr X) and y(t) (Corr Y) coordinates of cell trajectories was evaluated. This assumption is based on the idea that similar trajectories would have similar coordinates. For each cell line, a reference cell trajectory was selected, and all the other trajectories were used as a set of compared cell trajectories. The Pearson’s *r* between the reference coordinates and each one presented in the compared set (two by two) was calculated.

### Cell Spreading Area and Focal Adhesions

Cells were plated at a density of 2,000/cm^2^ on 23-mm glass dishes (Fluorodish, World Precision Instrument) and PAAm substrates. Cells were fixed and immunostained to evaluate the spreading area at 24 h from seeding. Cells were washed twice in PBS, fixed in 4% paraformaldehyde (Sigma Aldrich) for 20 min, and then rinsed twice with PBS and permeabilized in 0.3% Triton X-100 (Sigma-Aldrich) for 5 min. Cells were washed three times in PBS and blocked for 15 min in 10% bovine serum albumin (BSA, Sigma-Aldrich) and then incubated for 1 h with rabbit monoclonal paxillin-antibody (Abcam, ab32084) at a dilution of 1:200 in 0.1% BSA-PBS. After washing in PBS, cells were incubated with Alexa 546 goat antirabbit secondary antibody (Life Technologies) and Alexa 488 phalloidin (Invitrogen) at a dilution of 1:200 in PBS for 1 h. Specimens were imaged using an Olympus IX81 inverted microscope and a 10x objective to quantify cell spreading area. Fluorescent images were imported into ImageJ software (NIH, Bethesda, MD, United States) for post-processing, analysis, and estimation of cell area. Images of all single cells were thresholded manually based on the actin staining and then the area of the cell body was calculated. For focal adhesion (FA) analysis, cell images were acquired by a confocal microscope SP5 (Leica) equipped with a 25× water immersion objective plus 4 × magnification of digital zoom. The image size was set equal to 2,048 × 2,048 pixels with a pixel size of 76 nm. To quantify FA length, the paxillin images were assembled into a stack. First the stack was Gaussian-filtered using a radius of 20 pixels. This stack was then subtracted from the original stack to reduce diffuse background signal and adhesions were measured by thresholding the stacks and using an ellipse-fitting function in ImageJ.

### Atomic Force Microscopy to Study Cell Mechanics

The mechanical properties of mesothelial cells cultured on glass and PAAm substrates were studied using a commercial AFM (Nanowizard II, JPK Instruments, Germany). AFM is combined with an optical inverted microscope (Zeiss) that allows the precise lateral positioning of the AFM tip over cells. To test cell mechanics, a tipless silicon nitride cantilever (MLCT-O10, Bruker AFM Probes) with a triangular shape was used. A spherical polystyrene tip (6 µm, Sigma-Aldrich) was glued onto the front of the tipless cantilever using an optical adhesive (NOA63, Norland). Cantilever spring constant (0.07 N/m) was determined from thermal fluctuation before experimentation. At least 10 square arrays of 8 × 8 indentations, covering (1 × 1) μm^2^ areas of cells or gels, were performed to quantify the stiffness (Young’s modulus). To test cell mechanical properties, measurements were conducted in cell culture medium supplemented with 12.5 mM HEPES buffer (EuroClone) at 37°C. To investigate the dependence of cell elasticity on cellular density, experiments were performed on both single cells and cell monolayer cultured on glass. Cells were indented approximately on the cytoplasmic region. The indentation depth was chosen to be 50 nm. The force indentation curves from each measurement were analyzed using a Hertzian model to obtain the Young’s modulus of each cell. In fact, the Hertz model gives the following relation between the indentation δ and the loading force F in the case of an infinitely hard sphere of radius R (AFM tip) touching a soft planar surface:

$$F_{sphere}=\frac{4}{3}\frac{E}{\left(1-\nu \right)}\sqrt{R}\delta^{\frac{3}{2}}$$

where E is the Young’s modulus and ν is the Poisson’s ratio, which, for biological material, is generally set to 0.5 (incompressible materials).

### Statistical Analysis

Data are reported as mean ± standard error of the mean (SEM), unless otherwise indicated. Statistical comparisons were performed with a Student’s unpaired test when data exhibit a normal distribution. Otherwise, a nonparametric Kruskal–Wallis test was used. *p* values <0.05 denote statistical significance.

## Results

### Cell Proliferation and Migration

**FIGURE 1:**
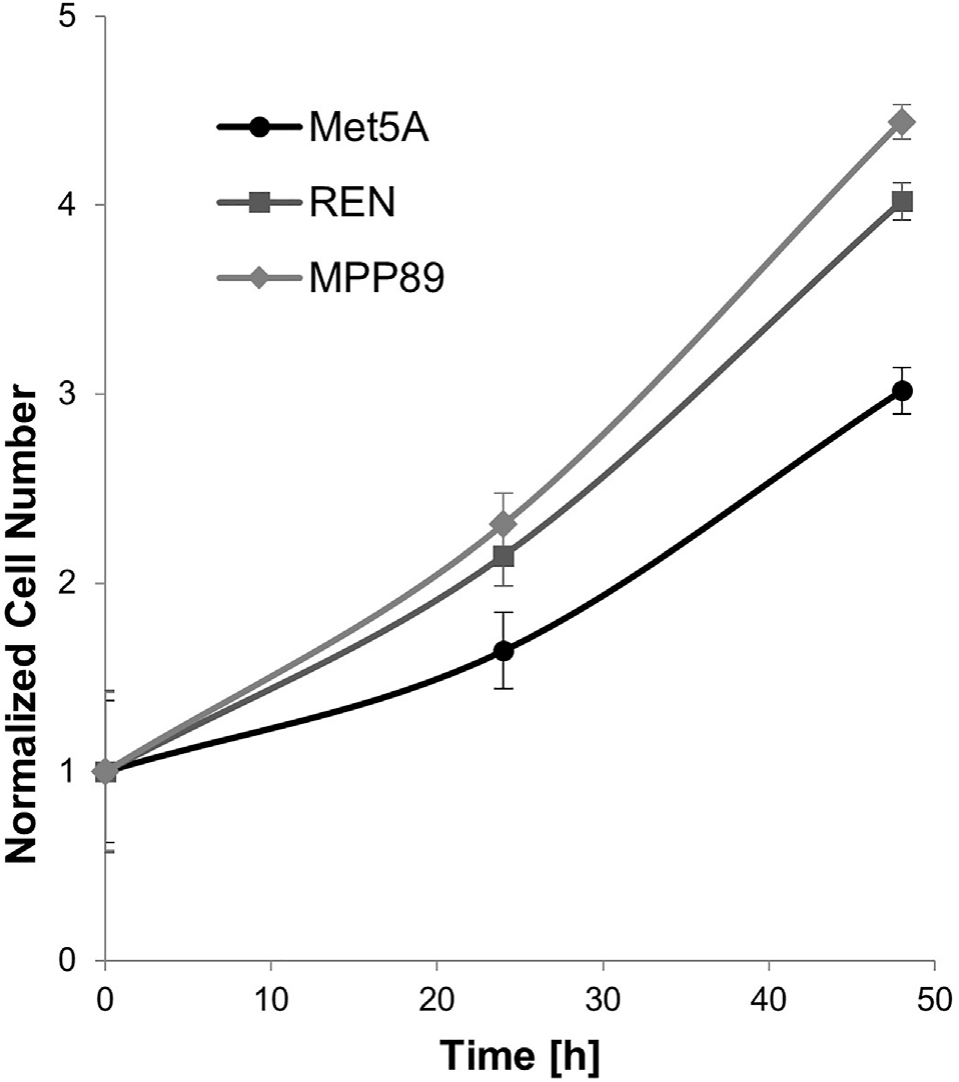
Cell proliferation was monitored for 48 h. Proliferative capacity of malignant cell lines was significantly higher than benign Met5A.

Cell proliferation and migration are considered two fundamental parameters to describe the aggressiveness of tumor cells. Then, the proliferation of normal mesothelial cells and MPM cell lines was analyzed at 24 and 48 h from cell seeding. As expected, both MPP89 and REN malignant cell lines exhibited higher proliferation compared to the benign one, but only a slight difference in the proliferation capability was observed between MPP89 and REN cells (Figure 1). Cell number of Met5A, REN, and MPP89 increased respectively by 64%, 114%, and 132% at 24 h from cell culture and by 202%, 302%, and 344% at 48 h. The proliferation rate of Met5A and MPP89 lines also became dependent on the substrate stiffness, as reported in Supplementary Figure S1, when evaluated on PAAm hydrogels with Young’s moduli of 0.3, 4, and 30 kPa.

**FIGURE 2:**
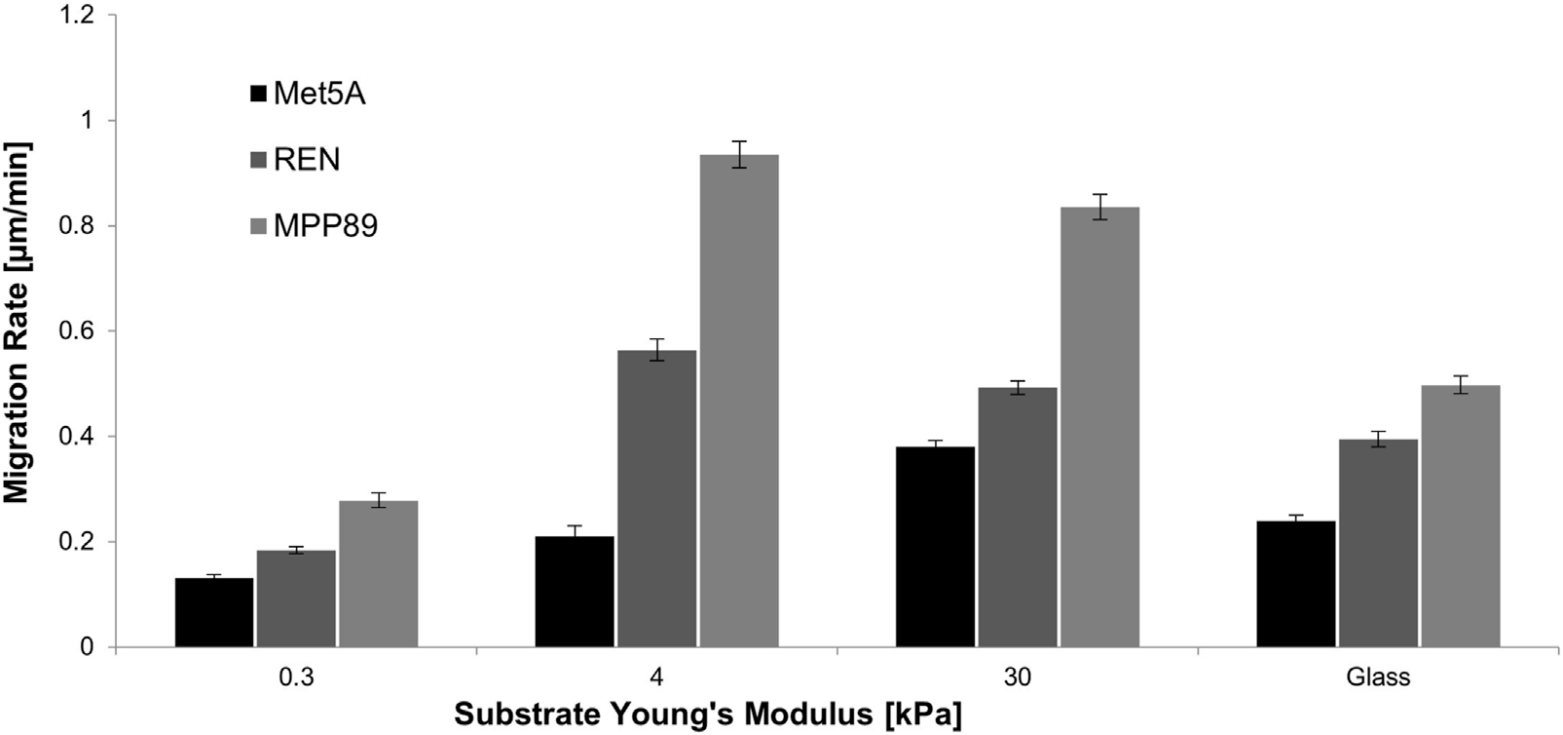
Single-cell migration rate on PAAm substrates and glass dishes. On glass, MPM cells exhibited significantly higher migration rate compared to benign cells. Moreover, MPP89 became significantly faster than REN cells. On PAAm substrates, MPM cells exhibited increasing motility compared to the healthy cell line, except for the case of 0.3 kPa, where REN migration was approximately the same as the healthy counterpart. Results were presented as mean ± S.E.M.

The influence of such stiffness was then also investigated on single-cell migration experiments, conducted on PAAm and on glass dishes (Fluoro-dish, World Precision Instruments, FD35–100). Results obtained from quantitative time-lapse microscopy revealed that, independently from substrate stiffness, both tumor cell lines displayed an increased motility compared to the healthy cell line (Figure 2; Table 1). MPP89 cells, instead, became always faster than REN independently from substrate stiffness. Of interest, the migration results showed a biphasic migration-velocity dependence on substrate stiffness for MP89 and REN cell lines, reaching the maximum velocity on 4-kPa hydrogels (Figure 2; Table 1). In particular, REN and MPP89 cells reached the maximum velocity on 4-kPa hydrogels and Met5A cells on 30 kPa, even. Considering the very big difference between the maximum velocity of benign and malignant cells, we tested cell migration also for an additional stiffness of 13 kPa finding for it a maximum in velocity values (Supplementary Figure S2), confirming the biphasic behavior observed for REN and MP89 cells.

**TABLE 1:** Statistical analysis for data of single cell migration. ***p < 0.001, **p < 0.01, NS—not significant.

			Met5A				REN				MPP89			
			Substrate young’s modulus [kPa]											
			0.3	4	30	Glass	0.3	4	30	Glass	0.3	4	30	Glass
Met5A	Substrate young’s modulus [kPa]	0.3	-	***	***	***	***	***	***	***	***	***	***	***
		4	-	-	***	NS	NS	***	***	***	**	***	***	***
		30	-	-	-	***	***	***	***	NS	***	***	***	***
		Glass	-	-	-	-	**	***	***	***	NS	***	***	***
REN		0.3	-	-	-	-	-	***	***	***	***	***	***	***
		4	-	-	-	-	-	-	**	***	***	***	***	***
		30	-	-	-	-	-	-	-	***	***	***	***	NS
		Glass	-	-	-	-	-	-	-	-	***	***	***	***
MPP89		0.3	-	-	-	-	-	-	-	-	-	***	***	***
		4	-	-	-	-	-	-	-	-	-	-	**	***
		30	-	-	-	-	-	-	-	-	-	-	-	***

**FIGURE 3:**
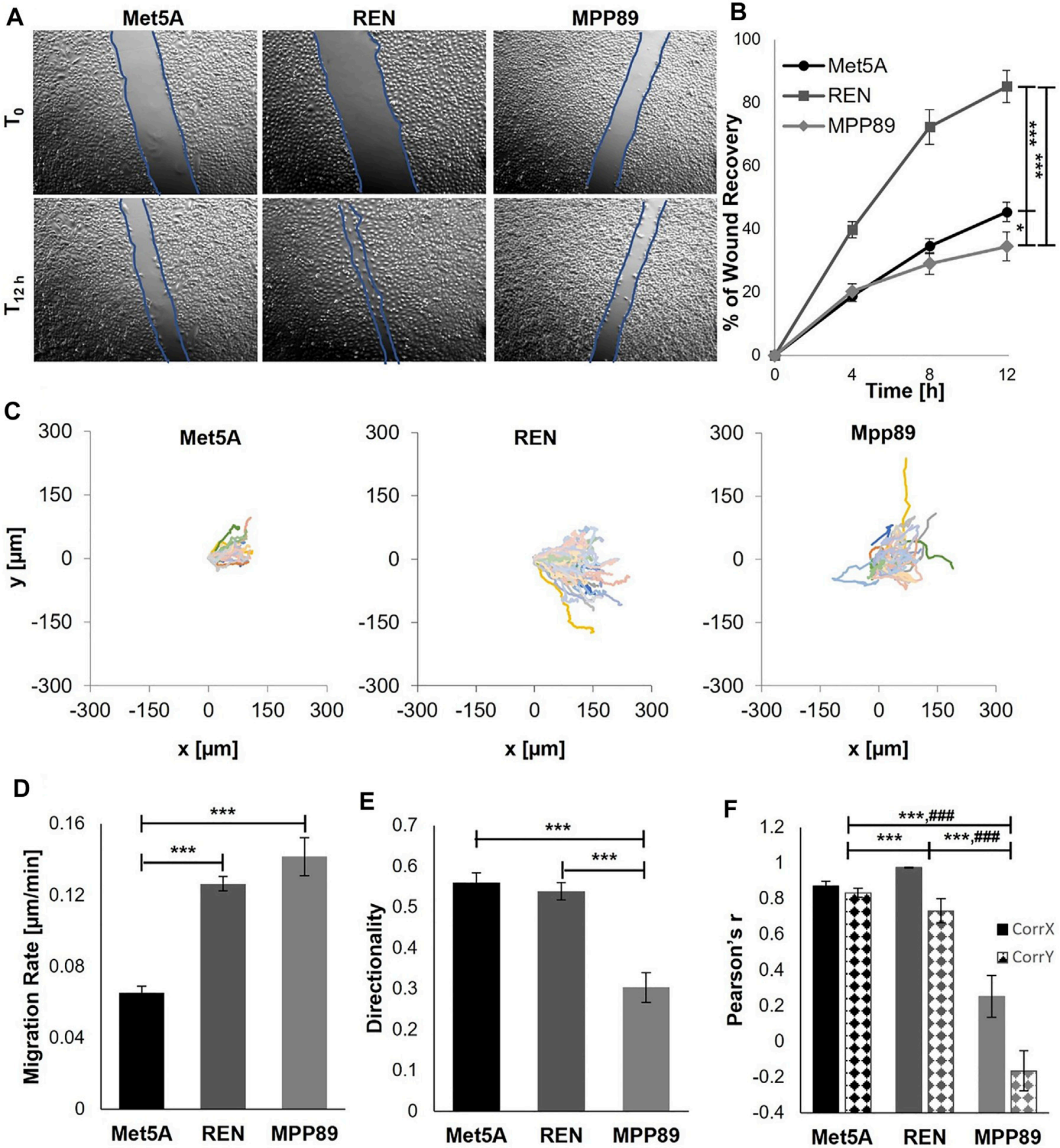
Collective migration analyzed in terms of wound percentage reduction in wound size **(A,B)**. REN cells closed the wound approximately after 12 h, whereas Met5A and MPP89 were not able to close the wound in the investigated interval of time. Plot at origin of trajectories of Met5A (left), REN (middle), and MPP89 (right) cells at the far end of the wounds **(C)**. Trajectories of cells were obtained by manual tracking using ImageJ and Manual Tracking plugin (http://rsweb.nih.gov/ij/). Migration velocity **(D)**, directionality **(E)**, and the Pearson correlation coefficient (Pearson’s *r*) between the x(t) (Corr X) and y(t) (Corr Y) coordinates of cell trajectories **(F)** at the far ends of the wound **(F)**. Results were presented as mean ± S.E.M. ***p < 0.001, **p < 0.01, *p < 0.05 **(B,D,E)**, ***p < 0.001 statistical analysis referred to Corr X data, ###*p* < 0.001 statistical analysis referred to Corr Y data **(F)**.

Collective migration on glass was then analyzed through a wound healing assay of 12 h. Cells of benign control (Met5A) and MPP89 were not able to close the wound in the time interval investigated, while the wound was closed by REN cells approximately after 12 h (Figures 3A,B). MPP89 cells did not close the wound because they did not have the ability to migrate in a directional way and preferentially proliferated and migrated in lateral direction (scratch direction) rather than in the direction of wound closure. Moreover, migration velocity of single cells was calculated. Also in confluent conditions, malignant cell lines continued to show higher velocity than healthy ones (Figure 2; Figures 3C,D). The single-cell trajectories, at the far ends of the wound (Figure 3C), were also analyzed to calculate the migration rate, the directionality, and the Pearson correlation coefficient (Pearson’s *r*) between the x(t) (Corr X) and y(t) (Corr Y) coordinates (Figures 3E,F). The values of both Corr X and Corr Y in Met5A and REN cells were higher than 0.7, indicating a strong correlation. On the contrary, Corr X and Corr Y in MPP89 cells were equal to 0.2 and −0.1, indicating no correlation.

### Cell Morphological Changes and Mechanics

**FIGURE 4:**
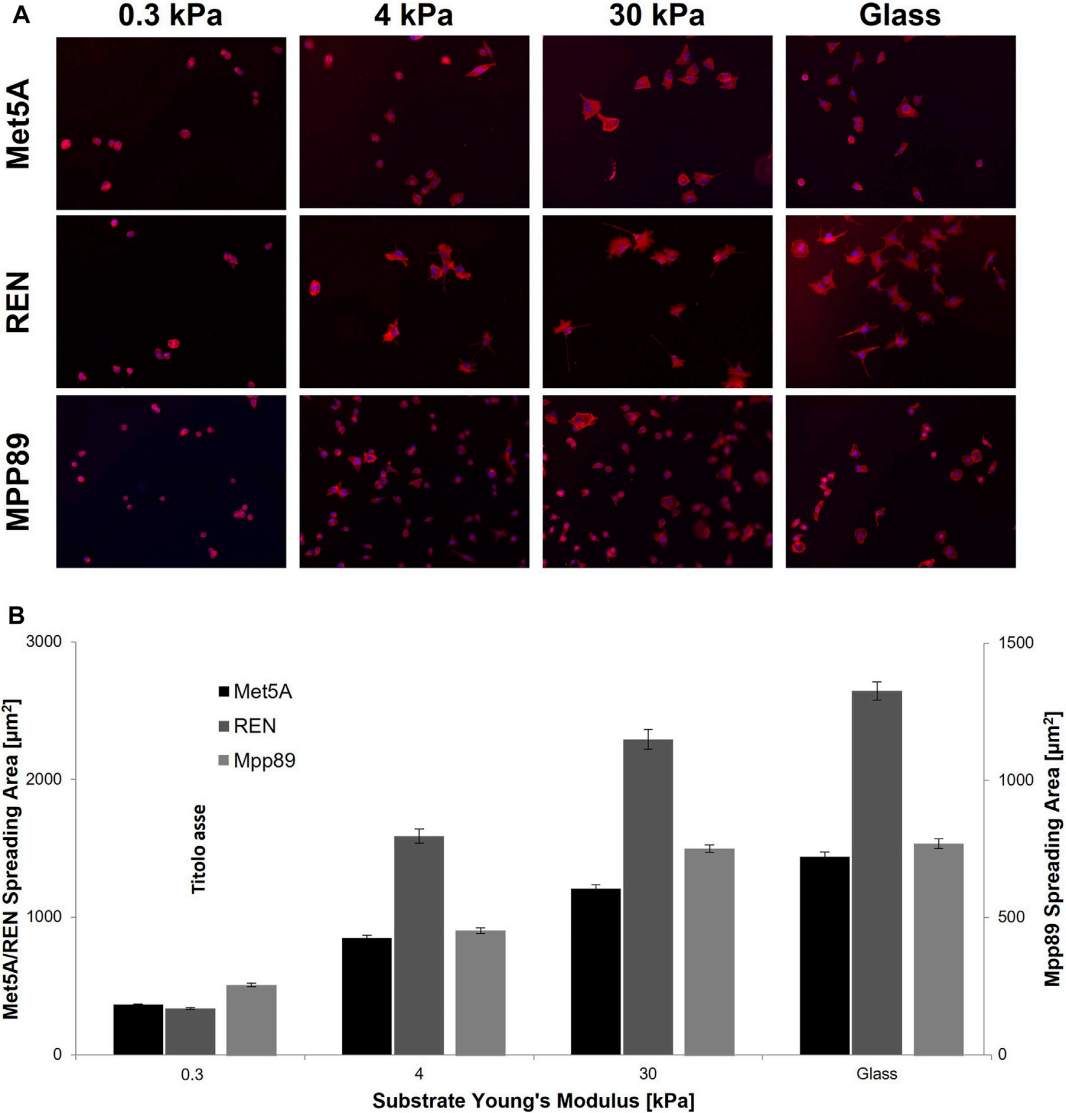
Cell spreading area on PAAm. Representative images of Met5A, REN and MPP89 cells cultured on PAAm and glass substrates **(A)**. Spreading area grew with the increase of substrate stiffness for all cell lines, except for MPP89 cells, whose area did not exhibit variation passing from 30 kPa to glass dishes **(B)**. Results were presented as mean ± S.E.M.

To evaluate the variation of morphological features of Met5A, MPP89, and REN cells in response to substrate stiffness, we quantified their spreading area when cultured on PAAm substrates of 0.3, 4, and 30 kPa and on glass dishes. Images were taken 24 h after seeding, to allow an optimal cell adhesion and spreading and only single cells (without contact with other adjacent cells) were analyzed. Unexpectedly, REN cells seeded on glass showed a wide spreading area, higher than MeT5A, whereas MPP89 exhibited the smallest spreading area (Figures 4A,B). Differently, on PAAm gels with increasing stiffness, cell area grew with a substrate stiffness for all cell lines, except for MPP89 cells, which did not exhibit variation passing from 30 kPa to glass (Figures 4A,B; Table 2). These results showed the preserved ability of both normal and malignant cells to sense matrix stiffness in terms of adhesion properties.

**TABLE 2:** Statistical analysis for data of single cell spreading. ***p < 0.001, NS—not significant.

			Met5A				REN				MPP89			
			Substrate young’s modulus [kPa]											
			0.3	4	30	Glass	0.3	4	30	Glass	0.3	4	30	Glass
Met5A	Substrate young’s modulus [kPa]	0.3	-	***	***	***	***	***	***	***	***	***	***	***
		4	-	-	***	***	***	***	***	***	***	***	***	***
		30	-	-	-	***	***	***	***	***	***	***	***	***
		Glass	-	-	-	-	***	***	***	***	***	***	***	***
REN		0.3	-	-	-	-	-	***	***	***	***	***	***	***
		4	-	-	-	-	-	-	***	***	***	***	***	***
		30	-	-	-	-	-	-	-	***	***	***	***	***
		Glass	-	-	-	-	-	-	-	-	***	***	***	***
MPP89		0.3	-	-	-	-	-	-	-	-	-	***	***	***
		4	-	-	-	-	-	-	-	-	-	-	***	***
		30	-	-	-	-	-	-	-	-	-	-	-	NS

**FIGURE 5:**
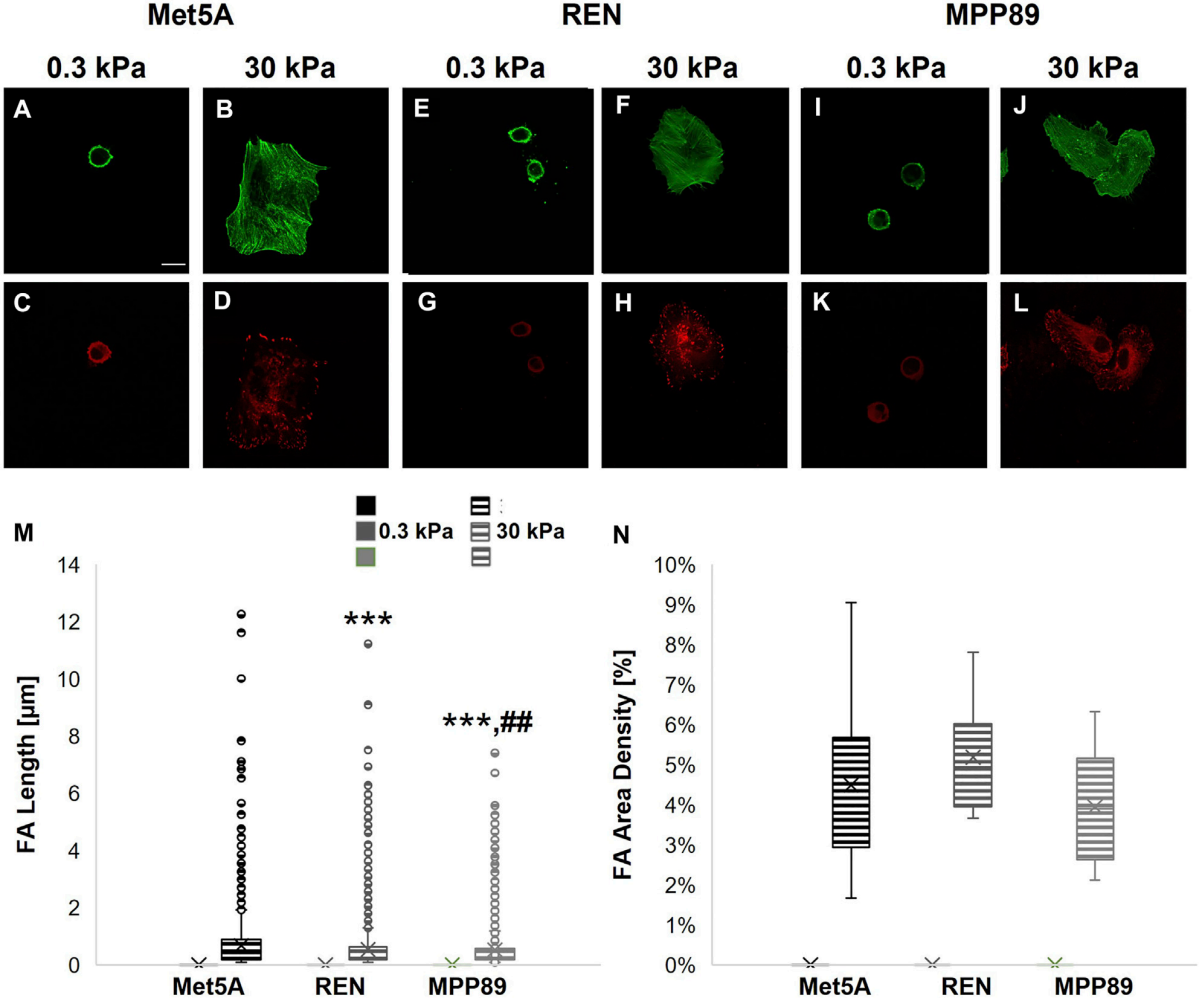
High-magnification images of cytoskeletal **(A,B,E,F,I,J)** and adhesion structures **(C,D,G,H,K,L)** on 0.3 and 3 kPa-PAAm substrates. Met5A **(A–D)**, REN **(E–H)**, and MPP89 **(I–L)** cells were stained for F-actin (green) and the FA protein paxillin (red). Bar, 20 μm. Assembled FAs were not observed on 0.3 kPa-PAAm and, consequently, their major length **(M)** and density **(N)** were not quantified on the softest substrate. Data **(M,N)** are presented as box plots (mean, median, interquartile range, and outliers). ***p < 0.001 with respect to Met5A on 30 kPa-PAAm substrate, ##p < 0.01 with respect to REN on 30 kPa-PAAm substrate.

**FIGURE 6:**
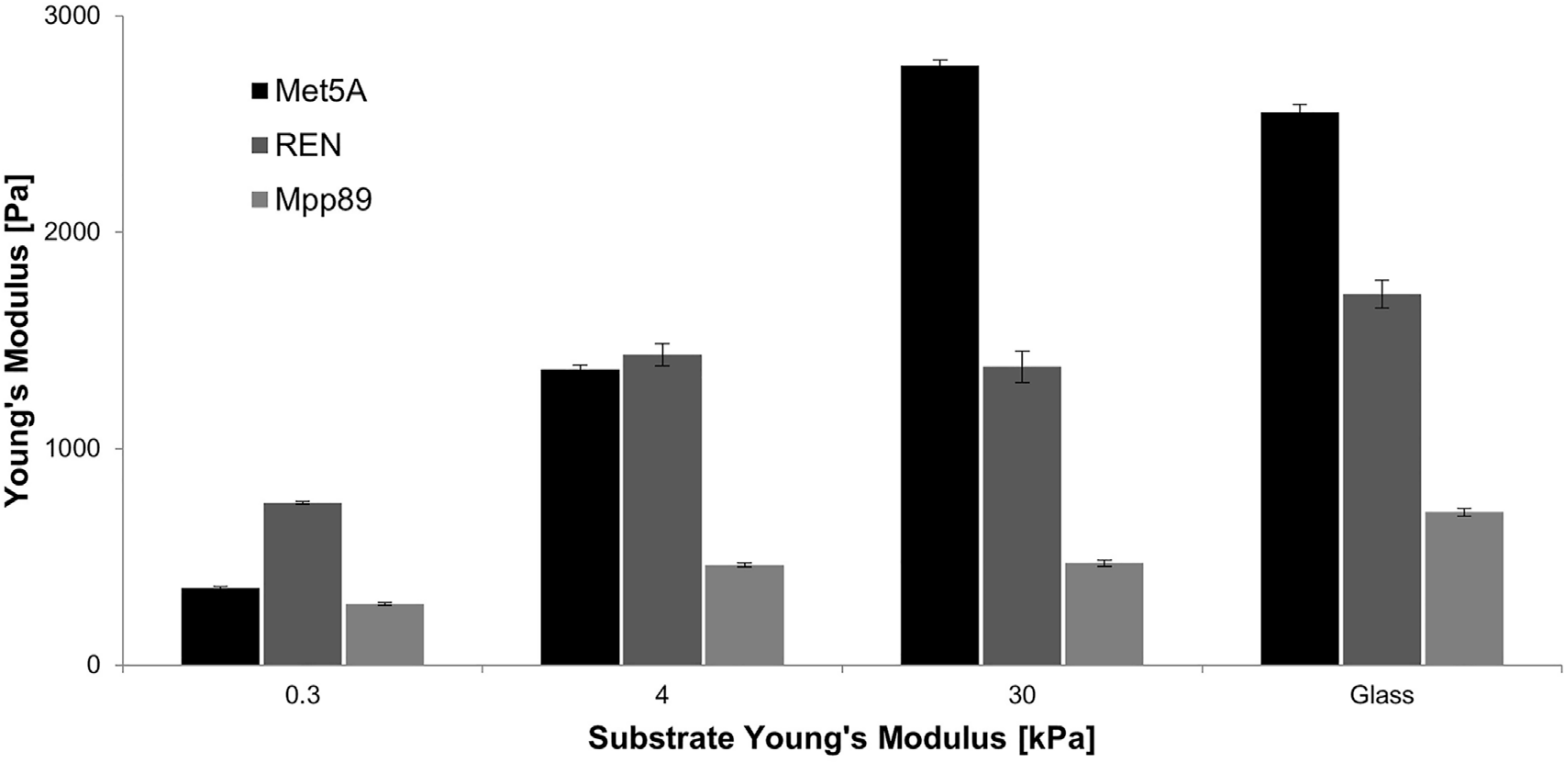
Apparent Young’s modulus for benign MET5a and malignant REN and MPP89 on PAAm and glass dishes. Met5A sensed variations in substrate stiffness changes, while MPP89 and REN cell lines did not feel difference in substrate stiffness passing from 4 kPa to glass. Results were presented as mean ± S.E.M.

Differences in cytoskeleton assembly as cells respond to the mechanical properties of substrate are reported in Figure 5A. F-Actin and paxillin were immunostained in the three cell lines investigated when seeded on PAAm substrates. The passage from 0.3 to 30 kPa is correlated to the aforementioned change in spreading area and, in turn, to the different architecture of F-Actin filaments. These became mainly organized and distributed cortically on the soft substrate (0.3 kPa) and assembled into bundles on the 30-kPa substrate, with decreasing length passing from Met5A and REN to MPP89 (Supplementary Figures S3, S4; Eltzner et al., 2015). No significant difference was found in F-actin density in the three cell lines, even if the mean and median values of this parameter show a decreasing trend passing from Met5A and REN to MPP89 cells (Supplementary Figure S4B). No assembling of FAs was detected on the 0.3-kPa substrate, but there was a decreasing concentration of cytosolic paxillin and actin cortex thickness passing from Met5A to REN and MPP89 (Supplementary Figure S3), whereas the box plots with length and density distribution of assembled FA on the stiffest PAAm are reported in Figure 5. Differently from cytosolic paxillin, FA length distribution became statistically different between the three cell lines. Cell mechanical properties were studied by AFM characterization. As a first step, we tested both single cells on glass to analyze the influence of cell density on mechanical properties. Benign cells exhibited higher Young’s moduli than malignant ones (a factor of about 3×, Figure 6; Table 3). Moreover, cancer cells showed reduction in stiffness with increasing metastatic potential. Cell mechanical properties were also evaluated on PAAm substrates and the results showed that while Met5A sensed variations in substrate stiffness in the entire range (0.3–30 kPa), MPP89 and REN cells no difference in their mechanics, similarly for REN spreading results, has been detected when passing from 4 to 30 kPa (Figure 6).

**TABLE 3:** Statistical analysis for data of single cell mechanics. ***p < 0.001, NS—not significant.

			Met5A				REN				MPP89			
			Substrate young’s modulus [kPa]											
			0.3	4	30	Glass	0.3	4	30	Glass	0.3	4	30	Glass
Met5A	Substrate young’s modulus [kPa]	0.3	-	***	***	***	***	***	***	***	***	***	***	***
		4	-	-	***	***	***	NS	NS	***	***	***	***	***
		30	-	-	-	*	***	***	***	***	***	***	***	***
		Glass	-	-	-	-	***	***	***	***	***	***	***	***
REN		0.3	-	-	-	-	-	***	***	***	***	***	***	***
		4	-	-	-	-	-	-	NS	***	***	***	***	***
		30	-	-	-	-	-	-	-	***	***	***	***	***
		Glass	-	-	-	-	-	-	-	-	***	***	***	***
MPP89		0.3	-	-	-	-	-	-	-	-	-	***	***	***
		4	-	-	-	-	-	-	-	-	-	-	NS	***
		30	-	-	-	-	-	-	-	-	-	-	-	***

## Discussion

MPM is a lethal cancer with increasing worldwide incidence. Unfortunately, it has a long latency period and, therefore, it is often diagnosed in the late stages, when its resistance to conventional chemo- and radiotherapy is very strong. For these reasons, the identification of new and specific biomarkers is of relevant importance to guarantee an early detection and diagnosis of MPM and, in turn, also to define more efficient treatments. To this purpose, a potential help could come from the study of cell mechanical properties that can be considered as a label free marker of cancer progression (Lee and Lim, 2007; Suresh 2007; Brunner et al., 2009). In fact, during cancer progression, cells transition from a fully mature, post-mitotic state to a proliferating motile cancerous state that involves a dramatic reorganization of the actin cytoskeleton and, consequently, a drastic change in their mechanical properties (Buda and Pignatelli, 2004; Lindberg et al., 2008; Ketene et al., 2012).

In this work, we studied the mechanical properties (Young’s modulus) of lung mesothelial cells, by using AFM. Biophysical characterization supports the analysis of biological behavior of cells, not only to discriminate malignant cells from benign ones, but also to identify the aggressiveness of tumor cells. It is well known that cancer cells are softer than their normal counterpart (Lekka et al., 2012). Although this phenomenon is well known, studying the correlation between cell mechanical properties and metastatic potential is an open question. At this aim, in this study, we proposed the mechanical characterization of a mesothelial cell line (Met5A) and two MPM cell lines (REN and MPP89) of different aggressiveness, which were not previously examined.

Tumorigenesis is accompanied by alterations in cell cytoskeletal structure that plays a critical role in cellular processes, including cell proliferation and migration activities, and influences cell shape, adhesiveness, and, consequently, mechanical properties (Pawlak and Helfman, 2001; Alberts et al., 2002). *In vitro* experiments on glass substrates revealed enhanced proliferative capacity of MPM cells. The invasiveness of MPM cells was also investigated using a wound healing scratch assay. We analyzed morphological and mechanical features of the proposed cell lines, and we discovered that morphology alone is not sufficient to discriminate malignant from benign cells. We found that REN cells appear morphologically similar to their heathy counterpart, and exhibit a great spreading area, about twice that of Met5A cells. Nevertheless, REN cells were more spread compared to MET5a; they appeared highly motile and able to repopulate the scratched area within 12 h. Furthermore, their mechanical properties became lower than those of healthy mesothelial cells, explaining their increased motility. Moreover, the analysis of biological and mechanical parameters could help to judge the aggressiveness of MPM. In fact, we noted that MPP89 cells present a very small spreading area, the ability to migrate rapidly, and low values of Young’s modulus, compared to REN cells. The most important difference between REN and MPP89 cells was in the way they closed scratched area in the wound assay. REN cells preserved their ability to heal wounds, while the wound healing process became seriously affected in the case of MPP89 cells. In fact, differently from Met5A and REN, migratory trajectories of MPP89 cells became completely uncorrelated, indicating that these cells gained the ability to detach from epithelial cell clusters and to move as single cells into a mesenchymal fashion.

Plastic and glass cell culture systems lack the properties required to mimic *in vivo* environments. Consequently, *in vitro* cultured cells generally have an altered behavior in terms of growth rate, morphology, and intracellular metabolic activities. In this context, it is of paramount importance to design biomaterials with micro-structural and mechanical properties able to organize cells and support a more *in vivo*-like cellular phenotype and behavior. In particular, it has been widely demonstrated that the stiffness of extracellular environment has a large impact, similar to chemical stimuli, on the regulation of cell behavior, in particular cell survival, proliferation, differentiation, and migration (Mason et al., 2012). For example, changes in the stiffness of glioma cells due to the rigidity of the substrate (Sen et al., 2009; Sen et al., 2012) and in the mechanical properties of cancer cells plated on soft collagen matrices have been reported (Staunton et al., 2016). It is important to study the role of ECM mechanics because of the changes in ECM composition and architecture in cancer. In fact, disease states are often accompanied by a local increase in ECM rigidity (Dean et al., 2005; Berry, 2006) due to local accumulation of a dense, crosslinked collagen matrix favoring detection of the tumor by physical palpation (Huang and Ingber, 2005; Levental et al., 2009). Cells that normally reside in a soft environment manifest enhanced proliferation and migration, and a loss of cell polarity, when cultured on stiffer matrices (Paszek et al., 2005). These aspects can be considered hallmarks of cancer cells, accompanying the transition from a relatively quiescent to a malignant phenotype, driven by local ECM remodeling and stiffening.

For these reasons, we also investigated how cellular functions and characteristics, such as cell migration, cell spreading area, and mechanics, were influenced by changes in substrate stiffness. To reproduce the environment that could better mimic the *in vivo* ECM, we had to consider that, in the body, tissue stiffness is not static, but changes during physiological processes, and in pathological responses like tumorigenesis. In particular, the elastic modulus of a normal human lung has been measured at 0.44–7.5 kPa, and this inhomogeneity depends in part on the region measured (alveolar wall, airway wall, or airway epithelium, for example) (White, 2015). However, in case of lung cancer, the elastic modulus grows above 15 kPa (Suki, 2014). The increase in the stiffness of the ECM can lead to phenotypic cellular changes such as increased proliferation and migration. The stiffness interval we investigated in this study encompassed lung physiological range in healthy and disease conditions. Previous studies agreed that normal cells have the ability, known as stiffness sensing (or mechanosensitivity), to detect and respond to the mechanical stiffness of the extracellular environment (Califano and Reinhart-King, 2010; Mason et al., 2012; Panzetta et al., 2020). Cancer cells do not show a one-way behavior. *In vitro* experiments on substrates of different stiffness demonstrated that only certain cancer cells exhibited a dependence on matrix rigidity for growth rate, spreading, and migration (Tilghman et al., 2010). In particular, rigidity-dependent cancer cells grew better on stiff/rigid matrices, and their lower growth rates, when plated on soft matrices, were caused at least in part by a selective alteration in cell cycle progression and by the induction of apoptosis. On rigid substrates, cell lines that demonstrated rigidity-dependent growth also spread extensively, formed prominent stress fibers and mature FAs, and migrated rapidly, while they appeared rounded and failed to productively migrate on less rigid gels (Ulrich et al., 2009; Tilghman et al., 2010; Panzetta et al., 2020). The regulation of growth in response to rigidity was controlled by FAK, ERK, and the small GTPase Rho expression (Paszek et al., 2005), or by an increase in cyclin D levels downstream of Rac activation (Klein et al., 2009). Rho GTPases and their downstream targets, which are critical mediators of cell spreading, migration, and contractility (Jaffe and Hall, 2005), may act as mechanosensory machinery that respond to the rigidity of the microenvironment.

In the present study, we found that matrix stiffness altered cytoskeletal and adhesion structures and mechanical properties of normal and MPM cells. The less structured cytoskeleton, the decreased migration rate, and the reduced Young’s modulus on soft substrates were also demonstrated for different lung cancer cell lines (Shukla et al., 2016). Indeed, our results support these findings and bring more knowledge on the effects of substrate stiffness on lung cancer cells. Both normal mesothelial and MPM cells reacted to substrate stiffening by increasing spreading area and mechanical properties and by showing biphasic migration-velocity dependence on substrate stiffness, with a peak value reached on 13-kPa substrates in the case of normal cells and on 4-kPa substrates in the case of MPM cells. Importantly, on the softest substrate, cancer cells show a significant reduction of migration rate, spreading area, cytoskeleton assembly, and Young’s moduli, until reaching values similar to those of normal cells. Recent works study how the normal stroma exerts tumor-suppressive signals to control tissue homeostasis (Weaver et al., 1997; Kaukonen et al., 2016; Panciera et al., 2020). It is demonstrated how the soft normal ECM can trigger the downregulation of cancer cell proliferation (56). Thus, our findings supported this new mechanism of ECM-mediated control of cancer cell behavior: by modulating ECM stiffness, cancer cell proliferation, migration rate, adhesion, and mechanical properties could be normalized.

In summary, we investigated, for the first time, the mechanical properties of MPM cells and their normal counterpart. Moreover, we have taken a first step in characterizing the response of MPM cell lines to changes in the rigidity of the surrounding microenvironment. Our findings showed that biophysical characterization of MPM cells appears to efficiently support the diagnosis of pleural effusions.

## Data Availability

The raw data supporting the conclusion of this article will be made available by the authors, without undue reservation.
