# Supplementary material for: ECM Mechanoregulation in Malignant Pleural Mesothelioma
Source: Front Bioeng Biotechnol. 2022 Feb 14;10:797900. doi: 10.3389/fbioe.2022.797900 (PMC8883334; doi:10.3389/fbioe.2022.797900)
Supplement: Supplementary file 1 [file DataSheet1.PDF]

Supplementary Material

ECM mechanoregulation in malignant pleural mesothelioma

Valeria Panzetta[1,2,3 †], Ida Musella[1,3 †], Sabato Fusco[4* #], Paolo A. Netti[1,2,3 #]

[1]Department of Chemical, Materials and Production Engineering, University of Naples Federico II, Naples, Italy

[2]Centro di Ricerca Interdipartimentale sui Biomateriali CRIB, University of Naples Federico II, Naples, Italy

[3]Istituto Italiano di Tecnologia, IIT@CRIB, Largo Barsanti e Matteucci, Naples, Italy

[4]Dipartimento di Medicina e Scienze della Salute "Vincenzo Tiberio", Università del Molise, Campobasso, Italy

*** Correspondence:**
Sabato Fusco
sabato.fusco@unimol.it

[†]These authors have contributed equally to this work and share first authorship
[#]These authors share last authorship

Supplementary Figures

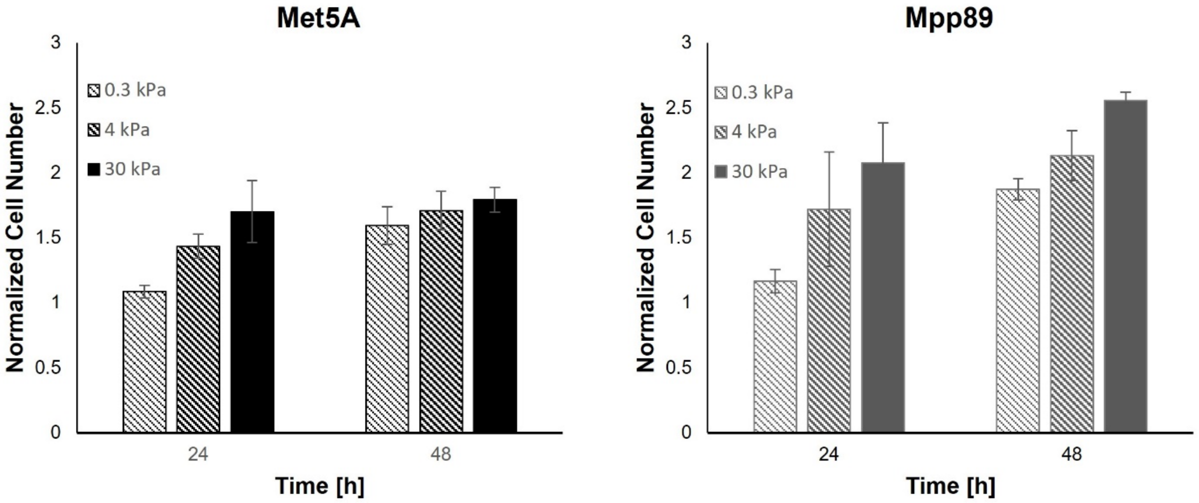

Supplementary Figure S1. Proliferation of Met5A (left) and Mpp89 (right) cell lines on PAAm substrates. Proliferation capacity grew with increasing of substrate stiffness for both cell lines, and as happens on glass dishes, malignant cells remained more proliferative than benign Met5A on all substrates.

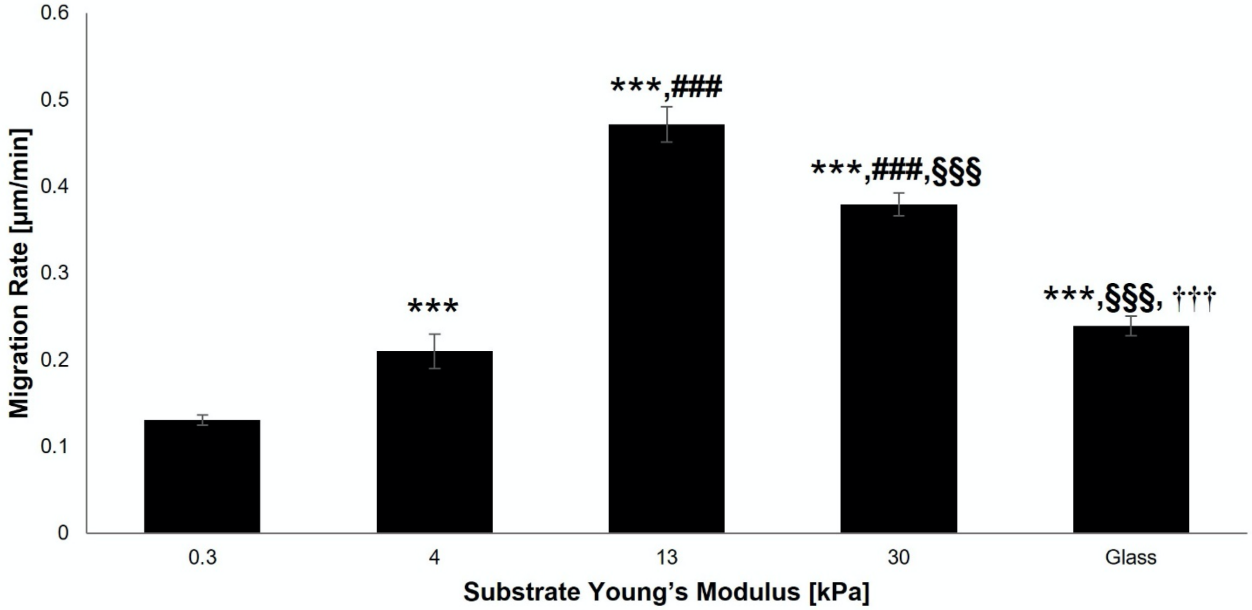

Supplementary Figure S2. Single cell migration rate of Met5A cells on PAAm substrates and glass dishes. Met5A cells reached the maximum velocity on 13 kPa hydrogels, exhibiting a biphasic behavior already observed for REN and MP89 cells (Figure 2). ***, $P < 0.001$ with respect to the 0.3 kPa substrate; ###, $P < 0.001$ with respect to the 4 kPa substrate; §§§, $P < 0.001$ with respect to the 13 kPa

substrate; †††, $P < 0.001$ with respect to the 30 kPa substrate. Data are reported as mean ± standard error (n > 98 for all substrate stiffness levels).

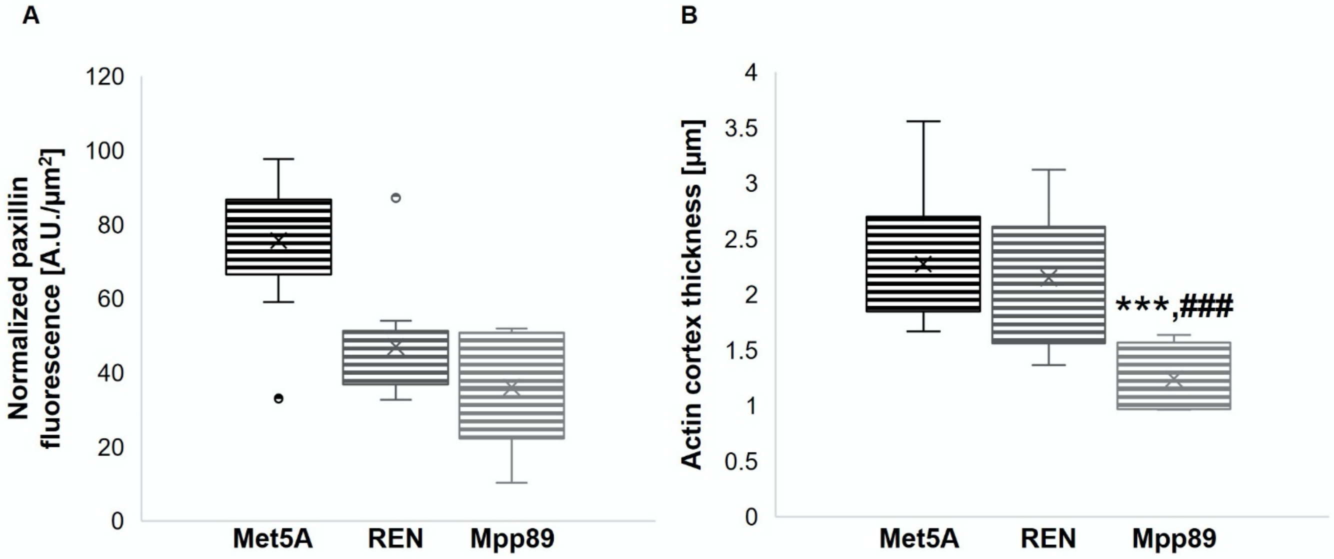

Supplementary Figure S3. Normalized fluorescence intensity of cytoplasmic paxillin (A) and actin cortex thickness (B) in Met5A (black), REN (grey) and Mpp89 (light grey) cells cultured on 0.3 kPa-PAAm subtsrates. Data are presented as box plots (mean, median, interquartile range, and outliers). *P<0.05, **P<0.01 and ***P<0.001 with respect to Met5A on 30 kPa-PAAm substrate, ###P<0.01 with respect to REN on 30 kPa-PAAm substrate.

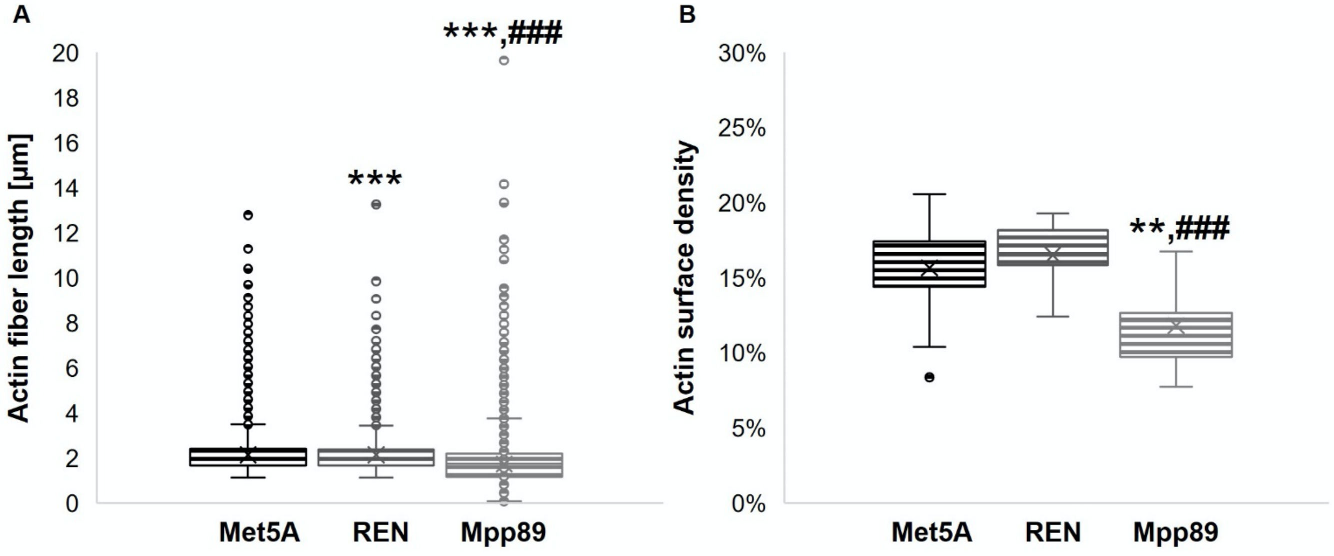

Supplementary Figure S4. The plugin Filament Sensor v0.1.7 was used to segment out single F-actin filaments from the fluorescence images (Eltzner et al., 2015). Images were filtered with a Gaussian filter of width 1 pixel and a subsequent Laplace filter of width 2 pixel. Moreover, only filaments starting from a minimal length of 20 pixel were considered. The F-actin density was calculated as ratio of total F-actin area to corresponding cell area. Actin fiber length (A) and actin density (B) in Met5A (black), REN (grey) and Mpp89 (light grey) cells cultured on 30 kPa-PAAm subtsrates. Data are presented as

box plots (mean, median, interquartile range, and outliers). *P<0.05, **P<0.01 and ***P<0.001 with respect to Met5A on 30 kPa-PAAm substrate, ###P<0.01 with respect to REN on 30 kPa-PAAm substrate.

References

Eltzner B, Wollnik C, Gottschlich C, Huckemann S, Rehfeldt F. The filament sensor for near real-time detection of cytoskeletal fiber structures. PloS one (2015) 1*0*(5), e0126346.
